# AlphaFold 3-Assisted Deciphering of the DNA Recognition by DREB1 Transcription Factors in Rice

**DOI:** 10.3390/ijms26136395

**Published:** 2025-07-02

**Authors:** Wenshu Wang, Wei Cai, Jiang Zhu, Yongsheng Zhu

**Affiliations:** 1 Institute of Crop Science of Wuhan Academy of Agricultural Sciences, Wuhan 430345, China; weicai@whu.edu.cn; 2 State Key Laboratory of Magnetic Resonance Spectroscopy and Imaging, Wuhan Institute of Physics and Mathematics, Innovation Academy for Precision Measurement Science and Technology, Chinese Academy of Sciences, Wuhan 430071, China; jiangzhu@wipm.ac.cn

**Keywords:** transcription regulation, abiotic stress, AP2 domain, structure modeling, C-repeat binding factor

## Abstract

Rice genome encodes ten OsDREB1 proteins that regulate tolerance to abiotic stresses such as cold and drought. OsDREB1s can bind to the C-repeat (CRT) element, dehydration response element (DRE), and GCC-box in gene promoters for transcription regulation. However, the recognition mechanism of OsDREB1s to these DNA elements remains unclear. Here, the structures of OsDREB1s were modelled using AlphaFold 3, which revealed a typical AP2 domain and a disordered KRP/RAGR motif adjacent to AP2 in all OsDREB1s. Structure modeling of OsDREB1A binding to CRT, DRE, and GCC-box showed that four Arg residues and a Glu (E66) from AP2 play important roles in binding to the major groove of DNA, while R40 in the KRP/RAGR motif was predicted to interact with the minor groove. The structure models revealed a few differences in the binding details for CRT, DRE, and GCC-box. Consistent with these predictions, OsDREB1A was evidenced to bind with the three DNA elements in slightly different affinities through EMSA experiments. Mutation analysis verified the key role of R40 and E66 in binding to CRT. Considering the highly conserved structure and sequence of the KRP/RAGR motif and AP2, we speculate that the DNA recognition mechanism found for OsDREB1A may be common for all OsDREB1s.

## 1. Introduction

Plants growing in nature usually face a variety of abiotic and biotic stresses. Abiotic stress factors such as temperature, drought, flood, light, salt, and heavy metals can affect plant growth and breeding to varied degrees and even lead to plant death in severe cases. Thus, plants have developed multiple mechanisms to adapt and survive in changing environments during evolution. Precise manipulation of the gene expression network in response to the abiotic stresses is one of the most important mechanisms. In doing so, many transcription factors are employed to specifically activate the expression of genes involved in stress resistance. Therein, dehydration response element binding proteins (DREBs) are the plant transcription factors broadly involved in regulating drought, high salt, cold, and heat responses [1].

DREB transcription factors belong to the AP2/ERF superfamily which is named due to the intrinsic AP2 domain [2], and can be further divided into two groups, DREB1 and DREB2 [3,4,5,6]. DREB1s, also known as the C-repeat binding factors (CBFs), were firstly recognized by their role in regulating cold response by activating the expression of cold-responsive (COR) genes in Arabidopsis [7,8,9,10,11], and further their roles in drought, high salt, and heat responses were revealed [12,13,14]. DREB1s can bind to the C-repeat (CRT) element (GCCGAC), dehydration response element (DRE) (ACCGAC), and GCC-box (GCCGCC) with their AP2 domains to target downstream genes [7,15,16]. A PKKPAGR motif adjacent to the N-terminal of AP2 of AtDREB1B (also termed AtCBF1) is also involved in DNA binding [17]. The region next to the C-terminal of AtDREB1B AP2 was suggested to function in trans-activation, and mutational analysis using truncation and alanine substitution revealed multiple hydrophobic motifs that contribute positively towards activation [18]. However, there is still a lack of structure–function relationship study on either the DNA binding or trans-activation of DREB1s.

The rice genome encodes ten CBF/DREB1 members, among which five are organized into two clusters (i.e., OsDREB1A, OsDREB1B, and OsDREB1H; OsDREB1I and OsDREB1J) in genome and the other five are dispersedly distributed (OsDREB1C, OsDREB1D, OsDREB1E, OsDREB1F, and OsDREB1G) [19]. Similar to the Arabidopsis homologs, rice DREB1s can regulate tolerance to multiple abiotic stresses. All investigated rice DREB1s including OsDREB1A/B/C/D/E/F/G manipulate plant cold tolerance [20,21,22,23,24,25,26,27]. Moreover, OsDREB1A/C/D/F/G affect high salt tolerance, OsDREB1C/E/G promote heat tolerance, and OsDREB1F/G regulate drought tolerance [20,22,23,25,28]. A remarkable study indicated that overexpression of OsDREB1C in rice can boost grain yield, confer higher nitrogen use efficiency, and promote early flowering [29]. These results suggest that genetically modulating of OsDREB1s in rice can achieve substantial benefits not only in abiotic stress resistance but also in grain yield.

The AP2/ERF superfamily has five subfamilies, namely AP2, DREB, ERF, RAV, and Soloist (a few unclassified proteins) [30]. The AP2 subfamily members have two tandem AP2 domains. The RAV subfamily members contain a B3 domain in addition to an AP2 domain. The members in the ERF and the DREB subfamily harbor only one AP2 domain. Typically, an AP2 domain has 60–70 residues, and is composed of a three-stranded β-sheet and an α-helix. The first structure of the AP2–DNA complex is that of Arabidopsis ERF1 AP2 in complex with a double-stranded DNA containing the GCC-box [31], which revealed that Arg and Trp residues in the β-sheet are important for DNA recognition. Later on, the structure of the AP2 from Arabidopsis ERF96 in complex with the GCC-box was solved [32]. In addition to the AP2 domain, an N-terminal α-helix in the flanking region of AtERF96 AP2 also interacts with DNA. In recent years, the structures of the AP2 and B3 domains from Arabidopsis TEMPRANILLO 1 (TEM1, RAV subfamily) and the two tandem AP2 domains from Arabidopsis WRINKLED1 (WRI1, AP2 subfamily) in complex with their respective target DNA sequence were independently solved [33,34], indicating a synergistic recognition of the target DNA by two DNA-binding domains in TEM1 and WRI1. However, the structure of OsDREB1 AP2 in complex with target DNA remains undetermined and the DNA recognition mechanism is still unknown.

Recently, an artificial intelligence system AlphaFold 3 with remarkably improved prediction accuracy for the protein–ligand complex structure is available to the scientific community, providing a convenient tool for predicting protein–DNA complexes [35]. In this study, we predicted the structures of apo OsDREB1s and the complexes of OsDREB1A DNA-binding domain (DBD) bound with CRT, DRE, and GCC-box using AlphaFold 3, respectively. The resulting structure models revealed that four Arg residues and a Glu residue located in the β-sheet of AP2 and conserved in both ERFs and DREB1s play important roles in binding to CRT, DRE, and GCC-box. In consistence with this, OsDREB1A DBD can bind with CRT, DRE, and GCC-box elements in slightly different affinities. Importantly, an Arg residue in the KRP/RAGR motif of OsDREB1A is crucial for the recognition of CRT and DRE elements, which is not found in ERFs. This is probably the reason why DREB1s can recognize the CRT and DRE elements, while ERFs cannot.

## 2. Results and Discussion

### 2.1. Structure Models of OsDREB1s

**Figure 1:**
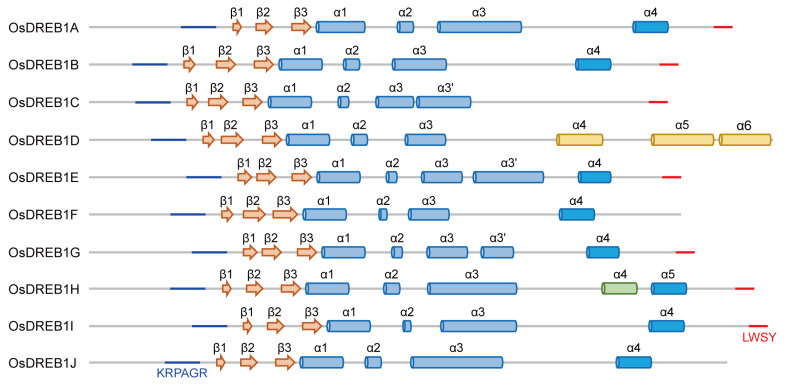
Structures of full-length OsDREB1s predicted by AlphaFold 3. The secondary structure elements of the ten members in the OsDREB1 family predicted by AlphaFold 3 are shown. The α1, α2, and α3 helices found in all members are colored in light blue. The α-helices adjacent to the C-terminal of α3 in OsDREB1C/E/G are labeled as α3′, as the total lengths of α3 and α3′ in the three members are similar to the α3 helices in OsDREB1A/H/I/J. The α-helix probably functioning in transcription activation is colored in marine blue. The α4, α5, and α6 unique to OsDREB1D are colored in light yellow. The α4 unique to OsDREB1H is colored in green. The disordered KRP/RAGR and LWSY motifs are shown in lines and colored in blue and red, respectively.

In order to understand the structural basis for the transcription regulation by ten OsDREB1s, we predicted their structure models using the AlphaFold 3 server. Sequences of full-length OsDREB1s were used as inputs (Table S1). The resulting secondary elements and 3D structure models of each OsDREB1 member are shown in Figure 1 and Figures S1–S10. The overall pTM values for full-length OsDREB1s are around 0.5. All OsDREB1s have an AP2 domain consisting of three β-strands and one α-helix (α1) with pIDDT values over 90, which indicate a high confidence level of the AP2 structural models. The N-terminal region adjacent to the AP2 domain is disordered in all OsDREB1s, while the C-terminal part is also largely disordered (including the LWSY motif), although there are a small number of α-helices scattered in this region. The N-terminal and C-terminal regions of OsDREB1s structural models show low pIDDT values, likely due to their low sequence complexity and limited similarity to proteins with known experimental structures.

**Figure 2:**
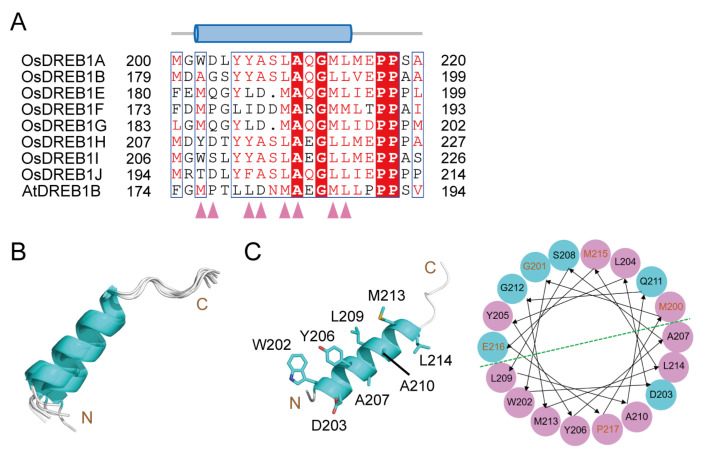
The conserved α-helix of OsDREB1s potentially functioning in transcription activation predicted by AlphaFold 3. (**A**) Sequence alignment of the potential transcription activation domain (TAD) of OsDREB1A/B/E/F/G/H/I/J and AtDREB1B with the secondary structure element of OsDREB1A shown on top. The residues at the hydrophobic side are labeled with pink arrows. (**B**) Superposition of the structures of potential TADs from OsDREB1A/B/E/F/G/H/I/J. (**C**) The helical structure and wheel representation of 18 residues (M200–P217) in OsDREB1A TAD (α4). The eight residues at the hydrophobic side are shown as sticks in cartoon view (left). In wheel representation (right), the hydrophobic side and hydrophilic side are separated by a green dashed line. Polar residues are shown in cyan circles, while nonpolar residues are in magenta circles. The residues in loops adjacent to α4 are in red font.

Eight OsDREB1s were predicted to contain a common α-helix (α4 in OsDREB1A/B/E/F/G/I/J and α5 in OsDREB1H), although the pIDDT values are low. The sequences of this common helix are similar to that of the identified transcription activation motif in AtDREB1B (Figure 2A) [18]. Thus, we speculate that this common α-helix may potentially function in transcription activation. This helix shows moderate sequence conservation in OsDREB1s and high consistence in length (Figure 2A,B). Analysis of the distribution of different amino acids revealed that these helices are amphipathic, as several hydrophobic amino-acid residues gather at one side of the helices (Figure 2C). It will be of great interest to verify the structure and supposed transcription activation function of this helix experimentally in the future. This helix is not found in OsDREB1C and has low sequence similarity in OsDREB1D (α4). On the other hand, OsDREB1D has two α-helices (α5 and α6) which are not found in other OsDREB1s. The distinctive C-terminal parts of OsDREB1C and OsDREB1D indicate that their functional manners may be different from that of other OsDREB1s in transcription regulation.

**Figure 3:**
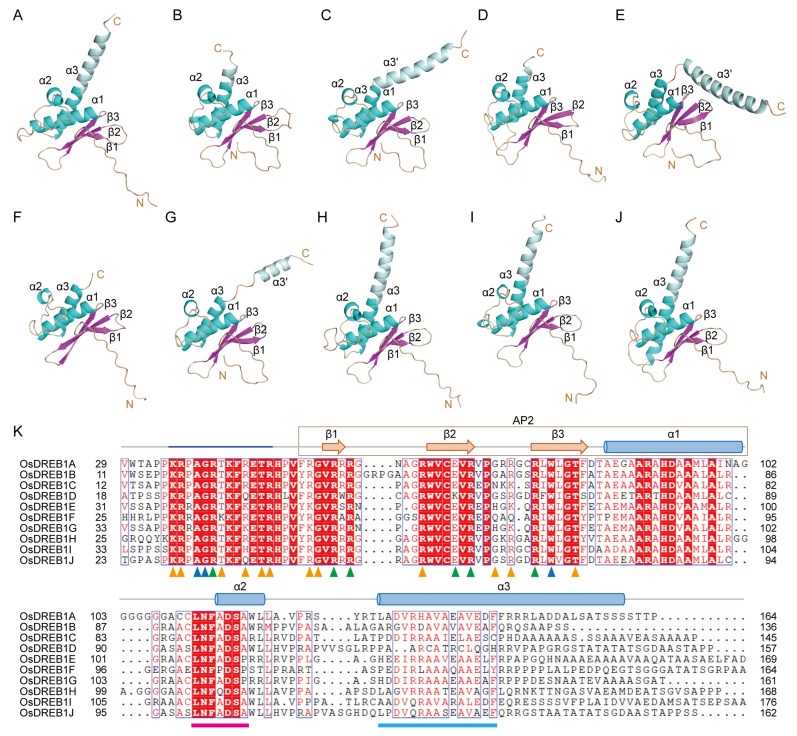
Structure and sequence features of the AP2 domains and adjacent regions of OsDREB1s. (**A**–**J**) Structures of the AP2 domain, the KRP/RAGR motif, and the α2 and α3 helices of OsDREB1s predicted by AlphaFold 3. The region not conserved in α3 (including α3′) is colored in pale cyan for distinction. (**K**) Sequence alignment of the AP2 domain, the KRP/RAGR motif, and the α2 and α3 helices of OsDREB1s with the secondary structure elements of OsDREB1A shown on top. The KRP/RAGR motif is marked in blue line. The residues predicted to form hydrogen bonds with DNA bases are labeled with green arrows. The residues interacting with DNA bases without hydrogen bond are labeled with marine blue arrows. The residues interacting with backbone phosphate groups are labeled with orange arrows. The LNFADSA/P motif is marked by magenta underline. The conserved region in α3 is marked by cyan underline.

In the AP2 domains, the three β-strands in each OsDREB1 member form an antiparallel β-sheet packed with α1, together comprising a typical AP2 structure with high pIDDT values (Figure 3A–J). The region adjacent to the N-terminus of the AP2 domain includes a disordered KRP/RAGR motif, similar to the PKKPAGR motif found in AtDREB1B [17]. Helices α2 and α3 adjacent to α1 pack to the AP2 domain in the structure models, which are found in all OsDREB1s, with the lengths and conformations of α3 varying a lot. The α2 contains part of a conserved LNFADSA/P motif (Figure 3K), corresponding to the previously described DSAWR motif in AtDREB1s [17]. The N-terminal half of α3 is also moderately conserved (Figure 3K), while the C-terminal part adopts various conformations in ten OsDREB1s. In OsDREB1C/E/G, this helix breaks into two parts, termed α3 and α3′ here. The pIDDT scores of α2 and α3 in all OsDREB1s are over 70 Figures S1–S10, suggesting a high confidence of the α-helix fold. However, whether α2 and α3 pack to the AP2 domain in 3D structure is still questionable. Moreover, the functions of α2 and α3 remain unknown, and no clue was obtained from the structure modeling currently.

### 2.2. DNA Recognition of OsDREB1A DBD

**Figure 4:**
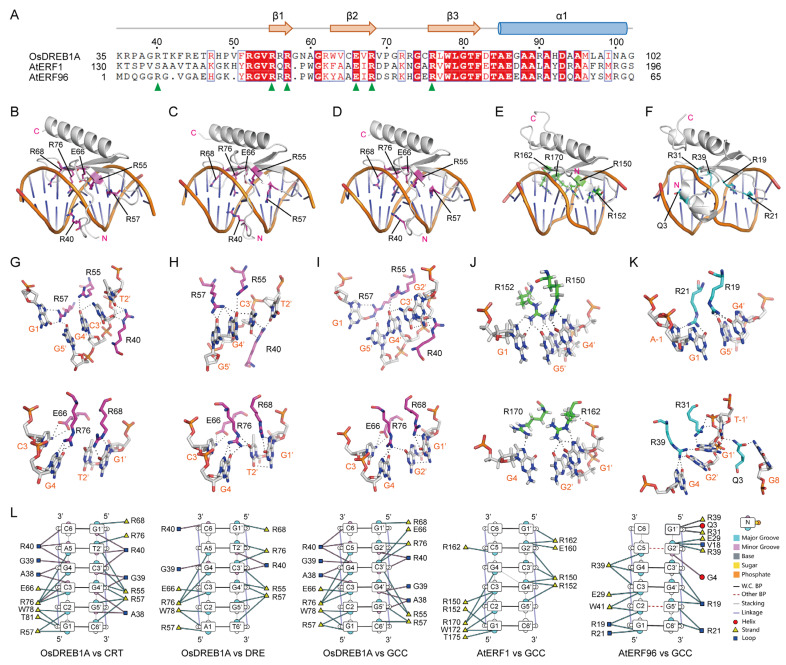
Recognition of CRT, DRE, and GCC-box elements by OsDREB1A DBD predicted by AlphaFold 3. (**A**) Sequence alignment of the DBDs of OsDREB1A, AtERF1, and AtERF96 with the secondary structure elements of OsDREB1A shown on top. The residues of OsDREB1A DBD predicted to form hydrogen bonds with DNA bases are labeled with green arrows. (**B**–**F**) The structure models of the complexes of OsDREB1A-DBD with CRT ((**B**), AlphaFold 3), OsDREB1A-DBD with DRE ((**C**), AlphaFold 3), OsDREB1A-DBD with GCC box ((**D**), AlphaFold 3), AtERF1 AP2 with GCC box ((**E**), PDB ID: 1GCC), and AtERF96 AP2 with GCC box ((**F**), PDB ID: 5WX9). The residues forming hydrogen bonds with DNA bases are shown as sticks. (**G**–**K**) The hydrogen bonds (black dashed lines) formed between amino acid residues and DNA bases in the protein–DNA complexes in (**B**–**F**). (**L**) Interaction patterns between amino acid residues and DNA bases in the protein–DNA complexes in (**B**–**F**) visualized by the DNAproDB server.

Compared to the members in the ERF subfamily of the AP2/ERF superfamily that recognize GCC-box (GCCGCC) DNA element, DREB1 subfamily members also recognize CRT (GCCGAC) and DRE (ACCGAC) elements which contain one-nucleotide (C5 to A5) substitution. To understand the underlying structural basis, we modeled the structures of the OsDREB1A AP2 domain complexed with dsDNA fragments containing CRT, DRE, and GCC-box elements, using AlphaFold 3, respectively. As the KRP/RAGR motif may also be involved in DNA binding, as hinted by AtDREB1B, it was included in the modeling (Figure 4A). The KRP/RAGR motif and AP2 domain of OsDREB1A are together termed the DNA-binding domain (DBD) hereafter. The resulting structure models have high confidence scores, with both ipTM and pTM over 0.8 (Figures S11–S13).

In these complexes, the structures of OsDREB1A-DBD show high consistency. The β-sheets bind to the major grooves of the DNA fragments, while the disordered KRP/RAGR motifs bind to the minor grooves (Figure 4B–D). These results support that the DBD of OsDREB1A contains both the AP2 domain and the KRP/RAGR motif. DNA-interacting residues are marked in the sequence alignment of AP2 domains and KRP/RAGR motifs from ten OsDREB1s (Figure 3K), which were found to be highly conserved. Six residues including R40 (KRP/RAGR motif), R55 (β1), R57 (β1), E66 (β2), R68 (β2), and R76 (β3) of OsDREB1A were predicted to form hydrogen bonds with the nucleotide bases of the DNA fragments, suggesting that they may play major roles in specifically recognizing the DNA sequences. The six residues are basically the same in ten OsDREB1s, except that E66 is substituted to a Lys residue in OsDREB1D. Twelve residues were predicted to interact with backbone phosphate groups with potentials to form hydrogen bonds (marked with orange arrows in Figure 3K). The hydrogen bond patterns predicted by AlphaFold 3 showed slight differences among the generated five models for each complex. A38 and W78 make hydrophobic interactions with the nucleotides. Considering the highly similar structures and conserved DNA-interacting residues of OsDREB1s, it is reasonable to believe that ten OsDREB1s recognize specific DNA elements in similar manners.

In comparison with the AP2 domains of AtERF1 and AtERF96 which have solved crystal structures complexed with the GCC-box element (Figure 4E,F), out of the six residues of OsDREB1A DBD predicted to form hydrogen bonds with the nucleotide bases, five from the β-sheet are identical in OsDREB1A, AtERF1 and AtERF96 (Figure 4A). As AtERF1 and AtERF96 do not have the KRP/RAGR motif, R40 in OsDREB1A has no counterpart in the two proteins. Although there is an Arg (R6) residue in N-terminus of AtERF96 AP2, it forms a hydrogen bond with the backbone phosphate group of DNA, not the base [32]. The six residues are involved in forming hydrogen bonds with DNA bases in all structures of OsDREB1A-DBD complexed with CRT, DRE, and GCC-box elements (Figure 4G–I). Four Arg residues in the β-sheet of AtERF1 and AtERF96 are involved in forming hydrogen bonds with bases of GCC-box elements, but the counterparts of OsDREB1A E66 do not (Figure 4J,K). An additional Glu (Q3) residue in the N-terminus of AtERF96 AP2 forms hydrogen bonds with G8 and T-1′ in the complementary chain, both adjacent to but not in the GCC-box.

Details for the core 6-bp nucleotide–amino acid interaction patterns of the five complexes are shown in Figure 4G–L. OsDREB1A-DBD, AtERF1-AP2 and AtERF96-AP2 bind to the GCC-box in similar patterns, wherein the four Arg residues (R55, R57, R68, and R76 in OsDREB1A) form hydrogen bonds with the six guanine bases (G1, G4, G1′, G2′, G4′, and G5′) from two DNA strands (Figure 4I–K). R40 of OsDREB1A was predicted to form hydrogen bonds with G2′ base and backbone sugar group of C3′, and E66 forms hydrogen bonds with C3 base, which are not found for AtERF1-AP2 and AtERF96-AP2. Thus, considering the modeling results, OsDREB1A-DBD may bind to GCC-box in a similar manner to AtERF1-AP2 and AtERF96-AP2.

Compared to the GCC-box, CRT and DRE have a substitution from the C5-G2′ base pair to an A5-T2′ base pair. R40 which forms a hydrogen bond with the G2′ base of GCC-box also forms a hydrogen bond with the T2′ base of DRE and CRT (Figure 4G–I). R76 which forms a hydrogen bond with the G2′ base of GCC-box also forms hydrogen bonds with the T2′ base of DRE, but not with the T2′ of CRT. Instead, R68 forms a hydrogen bond with the T2′ base of CRT. The difference between CRT and DRE is that there is a G1-C6′ base pair in CRT and an A1-T6′ base pair in DRE. R57 forms hydrogen bonds with bases of G4′ and G5′ in DRE instead of G1 in CRT and GCC-box. The roles of R55 and E66 are not altered for binding to the three DNA elements. From the modeling results, no remarkable difference for the bindings of OsDREB1A DBD to GCC-box, CRT, and DRE were found in terms of hydrogen bond number between DNA and protein, although the hydrogen bond patterns show differences. The hydrophobic interactions mediated by A38 and W78 show no remarkable differences in binding with the three DNA elements. Notably, the hydrogen bond patterns predicted by AlphaFold 3 altered to some extent in the generated five models for each complex, with those shown in Figure 4G–I appearing most frequently among the models, which therefore should be treated cautiously, although the confidence scores of modeling are high. Nevertheless, R40 undoubtedly makes an additional contribution to the binding in minor groove for OsDREB1A-DBD compared to AtERF1-AP2 and AtERF96-AP2 as predicted by AlphaFold 3. We speculate that the additional interaction between R40 and T2′ in CRT and DRE confers a higher affinity of OsDREB1A-DBD to the two elements than AtERF1-AP2 and AtERF96-AP2, which may be the reason why OsDREB1s recognize CRT and DRE, but ERFs do not.

### 2.3. Crucial Roles of R40 and E66 of OsDREB1A in Binding to CRT DNA Element

**Figure 5:**
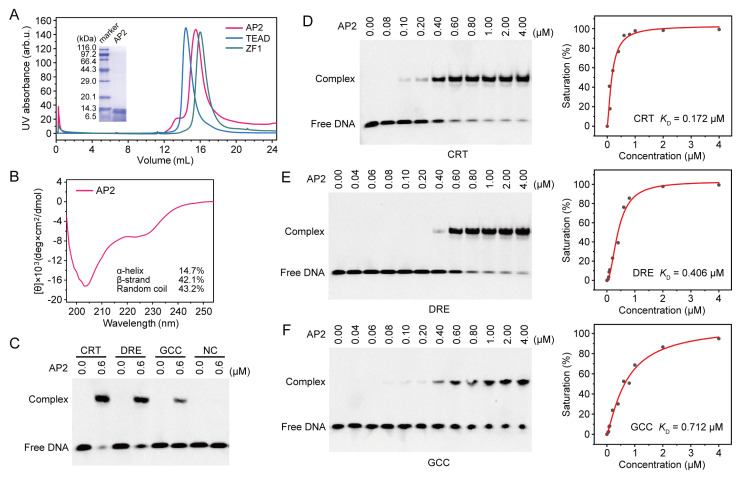
Binding affinities of OsDREB1A-DBD to CRT, DRE, and GCC-box DNA. (**A**) Size exclusion chromatography (SEC) of OsDREB1A-DBD during the third-round purification. SEC data of human TEAD1-TEAD and INSM1-ZF1 were used as controls. SDS-PAGE image of the purified OsDREB1A-DBD is shown as inset. (**B**) Circular dichroism spectrum of OsDREB1A-DBD. Secondary structure content calculated with CDNN software is indicated. (**C**) EMSA data using 0.01 μM FAM-labeled CRT, DRE, GCC-box, and negative control (NC) DNA to investigate the DNA binding of OsDREB1A-DBD. (**D**–**F**) EMSA data with gradient concentrations of OsDREB1A-DBD to estimate the binding affinities to CRT (**D**), DRE (**E**), and GCC-box (**F**) DNA. The gel images are shown on the left with the concentrations of OsDREB1A-DBD indicated on top. Curves and *K*_D_ values fitted from the EMSA data are shown on the right. The EMSA experiments were repeated three times with similar results.

In order to experimentally investigate the DNA recognition mechanism of OsDREB1A predicted by AlphaFold3, a truncated OsDREB1A-DBD protein (residue 32–103) sample containing the AP2 and the KRP/RAGR motif was obtained (Figure 5A). The elution volume of OsDREB1A-DBD (molecular weight 9.12 kDa) in size exclusion chromatography (SEC) analysis was approximately 15.5 mL, between the elution volume values of the TEAD domain of human TEAD1 protein (TEAD1-TEAD, molecular weight 10.49 kDa), and the ZF1 domain of INSM1 protein (INSM1-ZF1, molecular weight 5.75 kDa). Because TEAD1-TEAD and INSM1-ZF1 had been evidenced to exist as a monomer [36], it can be concluded that OsDREB1A-DBD also exists as a monomer. The circular dichroism (CD) spectrum of OsDREB1A-DBD showed two major negative peaks around 205 nm and 225 nm (Figure 5B). The secondary element content of OsDREB1A-DBD calculated according to the CD spectrum was basically similar to the predicted result by AlphaFold 3 (Figure 3K), supporting the disordered structure of the KRP/RAGR motif.

The binding of OsDREB1A-DBD to DNA fragments containing CRT, DRE, or GCC-box was respectively investigated using electrophoretic mobility shift assay (EMSA). The results showed that OsDREB1A-DBD can bind to CRT, DRE, and GCC-box elements but not the negative control DNA (Figure 5C). The band intensities of CRT, DRE, and GCC-box elements bound by OsDREB1A-DBD seemed to be slightly different. Thus, EMSA experiments with gradient concentrations of OsDREB1A-DBD were carried out and the binding affinity in term of dissociation constant (*K*_D_) was estimated for CRT, DRE, and GCC-box elements (Figure 5D–F). It was found that the *K*_D_ values of OsDREB1A-DBD for CRT, DRE, and GCC-box elements were approximately 0.172 μM, 0.406 μM, and 0.712 μM, respectively. OsDREB1A-DBD exhibits the highest affinity to CRT element. This may imply that G1 and A5 (T2′) are more welcome nucleotide bases of OsDREB1A-DBD at these positions.

**Figure 6:**
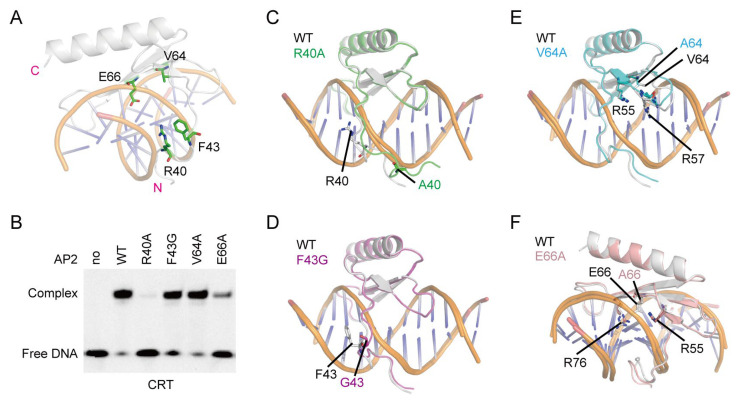
Key residues in the DNA recognition of OsDREB1A-DBD. (**A**) The structure model of the complex of OsDREB1A-DBD with CRT DNA predicted by AlphaFold 3. The residues selected for mutation analysis are shown as sticks. (**B**) EMSA data testing the binding of OsDREB1A-DBD and its mutants (final concentration of 0.6 μM) with CRT DNA. The EMSA experiments were repeated three times with similar results. (**C**–**F**) Superimposition of the structure models of wild-type OsDREB1A-DBD (white) and its mutants R40A (**C**), F43G (**D**), V64A (**E**), and E66A (**F**) in complex with CRT DNA generated by AlphaFold 3. The mutated residues and its adjacent residues are shown as sticks and marked.

Among the six residues of OsDREB1A DBD predicted to form hydrogen bonds with bases of CRT, DRE, and GCC-box elements, the roles of R55, R57, R68, and R76 are undoubtful, as the roles of their counterparts in AtERF96 for binding to DNA were evidenced by mutation analysis previously [32]. We investigated the key roles of R40 and E66 of OsDREB1A in DNA binding through mutation analysis (Figure 6A). Because the counterpart of F43 in AtDREB1B and that of V64 in AtDREB1A were shown to have important roles in DNA binding previously [15,17], the two residues were also selected for mutation analysis, although they both showed no interaction with CRT, DRE, and GCC-box elements in the complex structure models. EMSA experiments of the four mutants including R40A, F43G, V64A, and E66A with CRT DNA were carried out. The results showed that R40A has most serious impact on the binding of OsDREB1A-DBD to CRT DNA, and the binding was nearly abolished at the tested protein concentration (Figure 6B). E66A has moderate impact, while F43G and V64A almost have no effect on the binding with CRT DNA. To further interpret the results, we modeled the complex structures of the four mutants binding with CRT DNA (Figures S14–S17). The model of R40A showed that the R40-mutated KRP/RAGR motif disengages from the minor groove of DNA (Figure 6C), in consistence with the impaired DNA binding of R40A mutant in EMSA experiment. On the contrary, the models of F43G, V64A, and E66A did not show altered complex structure, although the orientations of R57 and R76 showed slightly changes (Figure 6D–F). Thus, F43 and V64 are not important for the binding of OsDREB1A-DBD to CRT. The impaired DNA binding of E66A may be due to the loss of the hydrogen bonds between E66 and the C3 base.

## 3. Materials and Methods

### 3.1. Structure Modeling and Analysis

Structure modeling of full-length OsDREB1s and the complexes of OsDREB1A DBD with different DNA elements was carried out using AlphaFold 3 [35]. The used sequences of proteins and DNA fragments are shown in Tables S1 and S2. The input information and quality scores of the resulting structure models including pIDDT, pTM, ipTM and expected position error are shown in Figures S1–S17 in the Supplementary Data file. Structure visualization and analysis were performed with PyMOL 1.3 software. The amino acid–DNA base interaction pattern was analyzed using DNAproDB [37]. Sequence alignment was conducted using Clustal Omega, adjusted based on structural superposition with PyMOL 1.3, and finally rendered using the ESPript 3 server [38].

### 3.2. Protein Expression and Purification

The DNA fragment encoding OsDREB1A DBD including the AP2 domain and the KRP/RAGR motif (residues 32–103) was cloned into the pET28 vector to be expressed with a C-terminal His-tag. The plasmids for expressing OsDREB1A-DBD mutants including R40A, F43G, V64A, and E66A were obtained through PCR-mediated mutagenesis of the wild-type (WT) OsDREB1A-DBD plasmid. All constructed plasmids were confirmed to be correct via Sanger sequencing and then transformed into *E. coli* BL21 (DE3) strain for protein production. For expressing different versions of OsDREB1A-DBD (WT and four mutants), the strains harboring corresponding plasmids were respectively inoculated using LB medium with shaking at 37 °C until the OD_600_ reached 0.6–0.8. Subsequently, isopropyl-β-D-thiogalactopyranoside (IPTG) was added to a final concentration of 0.2 mM to induce protein expression for 6 h at 37 °C. The *E. coli* cells were lysed in 50 mM NaH_2_PO_4_, 500 mM NaCl, 5 mM β-mercaptoethanol, at pH 6.8, using a high-pressure homogenizer (ATS Engineering, Suzhou, China). The supernatant was clarified by high-speed centrifugation and filtration, and then subjected to three-round purification using a HisTrap IMAC HP^TM^ column (5 mL, Cytiva, Marlborough, MA, USA), a HiTrap Heparin HP^TM^ column (5 mL, Cytiva), and a Superdex^TM^ 75 10/300 GL column (24 mL, Cytiva), respectively. The obtained proteins were concentrated to 50 μM in 50 mM NaH_2_PO_4_, 150 mM NaCl, 5 mM Dithiothreitol (DTT), at pH 6.8. The protein purity was evaluated by SDS-PAGE.

### 3.3. Circular Dichroism (CD)

The CD spectrum for OsDREB1A-DBD was recorded on a Chirascan^TM^ CD spectrometer (Applied Photophysics, Leatherhead, UK) from 195 to 260 nm with a step size of 1 nm and a bandwidth of 1 nm, using a 0.2 cm-path-length quartz cell, at 25 °C. Measurements were performed with OsDREB1A-DBD at 10 μM in 10 mM KH_2_PO_4_ (pH 7.0) three times. The averaged sample spectrum was subtracted with the spectrum of buffer solution to obtain the final spectrum. The secondary structure content was calculated using CDNN 1.0 software.

### 3.4. Electrophoretic Mobility Shift Assay (EMSA)

Double-stranded DNA (dsDNA) probes (CRT, DRE, GCC-box, and negative control) with 6-carboxy-fluorescein (FAM) labelled at 5′-end were obtained through annealing single-stranded DNA (ssDNA) oligos purchased from Sangon Biotech Co. (Shanghai, China) as described previously [36]. The sequences of used DNA probes are listed in Table S1. For each sample in the binding assay, dsDNA probe at a final concentration of 0.01 μM was mixed with the tested protein at the indicated concentration to set up a 10 μL mixture in 50 mM NaH_2_PO_4_, 150 mM NaCl, 5 mM DTT, 2% glycerol, at pH 6.8. The mixed samples were incubated for 15 min at room temperature, and then loaded into a 12% native PAGE gel in 0.5 × TBE (Tris-Borate-EDTA) buffer. After separation by electrophoresis, the gel was subjected to fluorescence detection by ChemiDoc MP instrument (Bio-rad, Hercules, CA, USA). The band intensity was quantified using Image Lab software, and the binding curves were fitted using Origin 2025 with the Equation (1).

(1) $$Y=\frac{B_{\max}X}{K_{D}+X}$$

## 4. Conclusions

In this study, the structures of full-length apo OsDREB1s and the complexes of OsDREB1A DBD bound by CRT, DRE, and GCC-box element were modelled using AlphaFold 3, respectively. The resulting structure models of apo OsDREB1s revealed a disordered KRP/RAGR motif and a typical AP2 fold in all OsDREB1s, which are also highly conserved in sequence. Complex structure models of OsDREB1A binding with three DNA elements suggested that the DNA-binding domain of OsDREB1s comprises of the KRP/RAGR motif and the AP2 domain, with KRP/RAGR motif binding in the minor groove and AP2 binding in the major groove of DNA. Four Arg residues and a Glu (E66 in OsDREB1A) residue located in the β-sheet of AP2 and conserved in both ERFs and DREB1s play roles in binding to CRT, DRE, and GCC-box in the major groove, through forming a hydrogen bond network. In consistence with this, OsDREB1A DBD can bind with CRT, DRE, and GCC-box elements as revealed by EMSA experiments, although in slightly different affinities. Importantly, an Arg residue (R40 in OsDREB1A) in the KRP/RAGR motif of OsDREB1s which is not found in ERFs was predicted to provide additional hydrogen bond interaction with the T2′ (paired to A5) base in the minor groove, although the hydrogen bond patterns was altered slightly in the generated five AlphaFold models. This is probably the reason why DREB1s can recognize the CRT and DRE elements, while ERFs cannot. Moreover, as R57 in the β-sheet prefers the G1 to A1 base in the DNA element, OsDREB1A DBD exhibits higher affinity to CRT than DRE. Mutation analysis with the EMSA experiment verified the key roles of R40 and E66 in binding to CRT DNA. The structure modeling by AlphaFold 3 also revealed a common amphipathic α-helix in the C-terminal part of eight OsDREB1s (OsDREB1A/B/E/F/G/H/I/J), potentially functioning in transcription activation. Taken together, this study presents structural insights into the recognition of CRT, DRE, and GCC-box elements by OsDREB1s, significantly updates the understanding of the transcription regulation by OsDREB1s, and provides information for manipulating crop gene expression network in response to abiotic stresses through gene editing, a potential approach for future applications.

## Data Availability

The original contributions presented in this study are included in the article/Supplementary Materials. Further inquiries can be directed to the corresponding author(s).

## Supplementary Materials

The supporting information can be downloaded at https://www.mdpi.com/article/10.3390/ijms26136395/s1.

## Abbreviations

The following abbreviations are used in this manuscript:

AP2	APETALA2
ERF	ethylene-responsive element binding factor
DREB	dehydration response element binding protein
CBF	C-repeat binding factor
RAV	related to ABI3/VP
CRT	C-repeat
DRE	dehydration response element
DBD	DNA-binding domain
PDB	Protein Data Bank
CD	circular dichroism
EMSA	electrophoretic mobility shift assay
FAM	6-carboxy-fluorescein
