# Supplementary material for: AlphaFold 3-Assisted Deciphering of the DNA Recognition by DREB1 Transcription Factors in Rice
Source: Int J Mol Sci. 2025 Jul 2;26(13):6395. doi: 10.3390/ijms26136395 (PMC12249877; doi:10.3390/ijms26136395)
Supplement: Supplementary file 1 [file ijms-26-06395-s001.zip › ijms-3674093-supplementary.pdf]

Table S1. Protein sequences of OsDREB1s used in this study.

Name	Sequence[a,b]
OsDREB1A	MCGIKQEMSGESSGSPCSSASAERQHQTVWTAPPKRPAGRTKFRETRHPVFRGVRRRGNAGRWVCEVRVPGRRGCRLWLGTFDTAEGAARAHDAAMLAINAGGGGGGGACCLNFADSAWLLAVPRSYRTLADVRHAVAEAVEDFFRRRLADDALSATSSSSTTPSTPRTDDDEESAATDGDESSSPASDLAFELDVLSDMGWDLYYASLAQGMLMEPPSAALGDDGDAILADVPLWSY
OsDREB1B	MEVEEAAYRTVWSEPPKRPAGRTKFRETRHPVYRGVRRRGGRPGAAGRWVCEVRVPGARGSRLWLGTFATAEAAARAHDAAALALRGRAACLNFADSAWRMPPVPASAALAGARGVRDAVAVAVEAFQRQSAAPSSPAETFANDGDEEEDNKDVLPVAAAEVFDAGAFELDDGFRFGGMDAGSYYASLAQGLLVEPPAAGAWWEDGELAGSDMPLWSY
OsDREB1C	MEYYEQEEYATVTSAPPKRPAGRTKFRETRHPVYRGVRRRGPAGRWVCEVREPNKKSRIWLGTFATAEAAARAHDVAALALRGRGACLNFADSARLLRVDPATLATPDDIRRAAIELAESCPHDAAAAAASSSAAAVEASAAAAPAMMMQYQDDMAATPSSYDYAYYGNMDFDQPSYYYDGMGGGGEYQSWQMDGDDDGGAGGYGGGDVTLWSY
OsDREB1D	MEKNTAASGQLMTSSAEATPSSPKRPAGRTKFQETRHLVFRGVRWRGCAGRWVCKVRVPGSRGDRFWIGTSDTAEETARTHDAAMLALCGASASLNFADSAWLLHVPRAPVVSGLRPPAARCATRCLQGHRRVPAPGRGSTATATATSGDAASTAPPSAPVLSAKQCEFIFLSSLDCWMLMSKLISSSRAKGSLCLRKNPISFCMVTNSYTALLLEYIILQMNSMIVLIHELSKYQVFLLLTMITHHLFQWRR
OsDREB1E	MEWAYYGSGYSSSGTPSPVGGDGDEDSYMTVSSAPPKRRAGRTKFKETRHPVYKGVRSRNPGRWVCEVREPHGKQRIWLGTFETAEMAARAHDVAAMALRGRAACLNFADSPRRLRVPPLGAGHEEIRRAAVEAAELFRPAPGQHNAAAEAAAAVAAQATAASAELFADFPCYPMDGLEFEMQGYLDMAQGMLIEPPPLAGQSTWAEEDYDCEVNLWSY
OsDREB1F	MDTEDTSSASSSSVSPPSSPGGGHHHRLPPKRRAGRKKFRETRHPVYRGVRARAGGSRWVCEVREPQAQARIWLGTYPTPEMAARAHDVAAIALRGERGAELNFPDSPSTLPRARTASPEDIRLAAAQAAELYRRPPPPLALPEDPQEGTSGGGATATSGRPAAVFVDEDAIFDMPGLIDDMARGMMLTPPAIGRSLDDWAAIDDDDDHYHMDYKLWMD
OsDREB1G	MDVSAALSSDYSSGTPSPVAADADDGSSAYMTVSSAPPKRRAGRTKFKETRHPVFKGVRRRNPGRWVCEVREPHGKQRIWLGTFETAEMAARAHDVAALALRGRAACLNFADSPRRLRVPPIGASHDDIRRAAAEAAEAFRPPPDESNAATEVAAAASGATNSNAEQFASHPYYEVMDDGLDLGMQGYLDMAQGMLIDPPPMAGDPAVGSGEDDNDGEVQLWSY
OsDREB1H	MDMAGHEVNSSSSSSGAESSSSSSGRQQYKKRPAGRTKFRETRHPVYRGVRRRGGAGRWVCEVRVPGKRGARLWLGTYVTAEAAARAHDAAMIALRGGAGGGGAACLNFQDSAWLLAVPPAAPSDLAGVRRAATEAVAGFLQRNKTTNGASVAEAMDEATSGVSAPPPLANNAGSSETPGPSSIDGTADTAAGAALDMFELDFFGEMDYDTYYASLAEGLLMEPPPAATALWDNGDEGADIALWSY
OsDREB1I	MCTSKLEEITGEWPPPALQAASTTSSSEPCRRLSPPSSKRPAGRTKFHETRHPVFRGVRRRGRAGRWVCEVRVPGRRGCRLWLGTFDAADAAARAHDAAMLALRGRAAACLNFADSAWLLAVPPPATLRCAADVQRAVARALEDFEQRESSSSVFPLAIDVVAEDAMSATSEPSAASDDDAVTSSSSTTDADEEASPFELDVVSDMGWSLYYASLAEGLLMEPPASGASSDDDDDAIVDSSDIADVSLWSY
OsDREB1J	MEKNTTAMGQLMSSSATTAATATGPASPKRPAGRTKFQETRHPVFRGVRRRGRAGRWVCEVRVPGSRGDRLWVGTFDTAEEAARAHDAAMLALCGASASLNFADSAWLLHVPRAPVASGHDQLPDVQRAASEAVAEFQRRGSTAATATATSGDAASTAPPSSSPVLSPNDDNASSASTPAVAAALDHGDMFGGMRTDLYFASLAQGLLIEPPPPPTTAEGFCDDEGCGGAEMELWS

[a] Sequences of AP2 domains are colored in red.

[b] Conserved sequences adjacent to N-terminal of AP2 domains are colored in blue.

Table S2. DNA sequences used in this study.

Name	Sequence[a]
CRT	F 5'-GTACGCCGACACT-3'<br>R 5'-AGTGTCGGCGTAC-3'
DRE	F 5'-GTATACCGACACT-3'<br>R 5'-AGTGTCGGTATAC-3'
GCC	F 5'-GTACGCCGCCACT-3'<br>R 5'-AGTGGCGGCGTAC-3'
Negative control (NC)	F 5′-AGTGGAATGTGCG-3′<br>R 5′-CGCACATTCCACT -3′

[a] The 6-bp core sequences of the DNA elements are underlined.

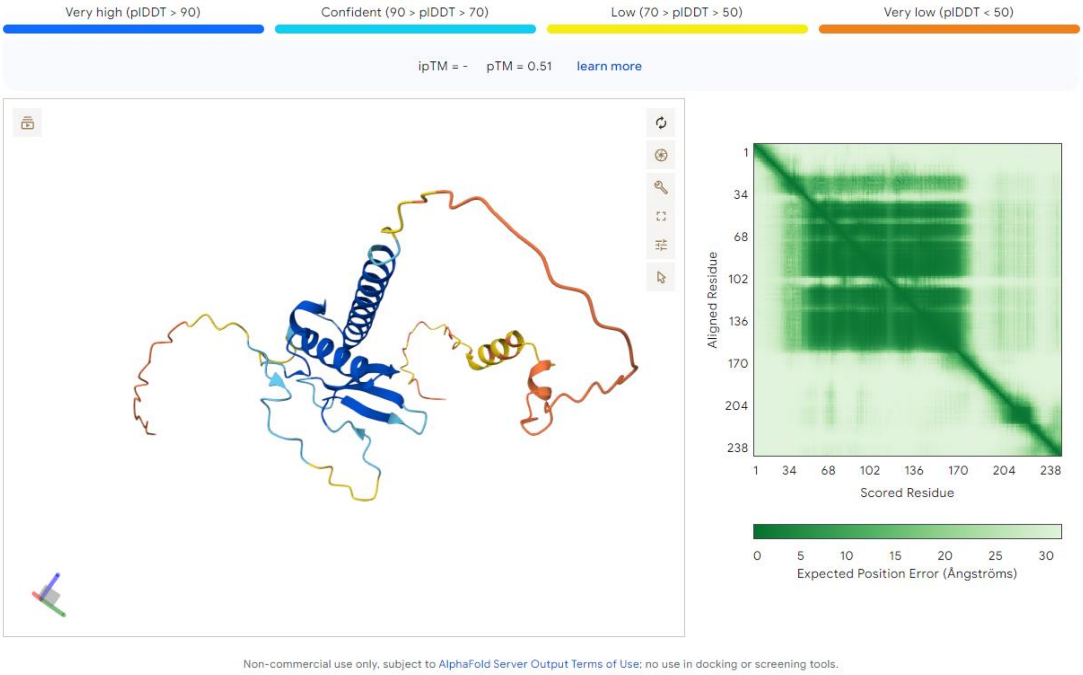

Information

Type	Copies	Sequence
Protein	1	(10) MCGIKQEMSG (20) ESSGSPCSSA (30) SAERQHQTVW (40) TAPPKRPAGR (50) TKFRETRHPV (60) FRGVRRRGNA (70) GRWVCEVRVP (80) GRRGCRLWLG (90) TFDTAEGAAR (100) AHDAAMLAIN (110) AGGGGGGGAC (120) CLNFADSAWL (130) LAVPRSYRTL (140) ADVRHAVAEA (150) VEDFFRRRLA (160) DDALSATSSS (170) STTPSTPRTD (180) DDEESAATDG (190) DESSSPASDL (200) AFELDVLSDM (210) GWDLYYASLA (220) QGMLMEPPSA (230) ALGDDGDAIL (238) ADVPLWSY

Seed: 2130785884

Figure S1. Quality and input information of the structure models of OsDREB1A generated using AlphaFold 3.

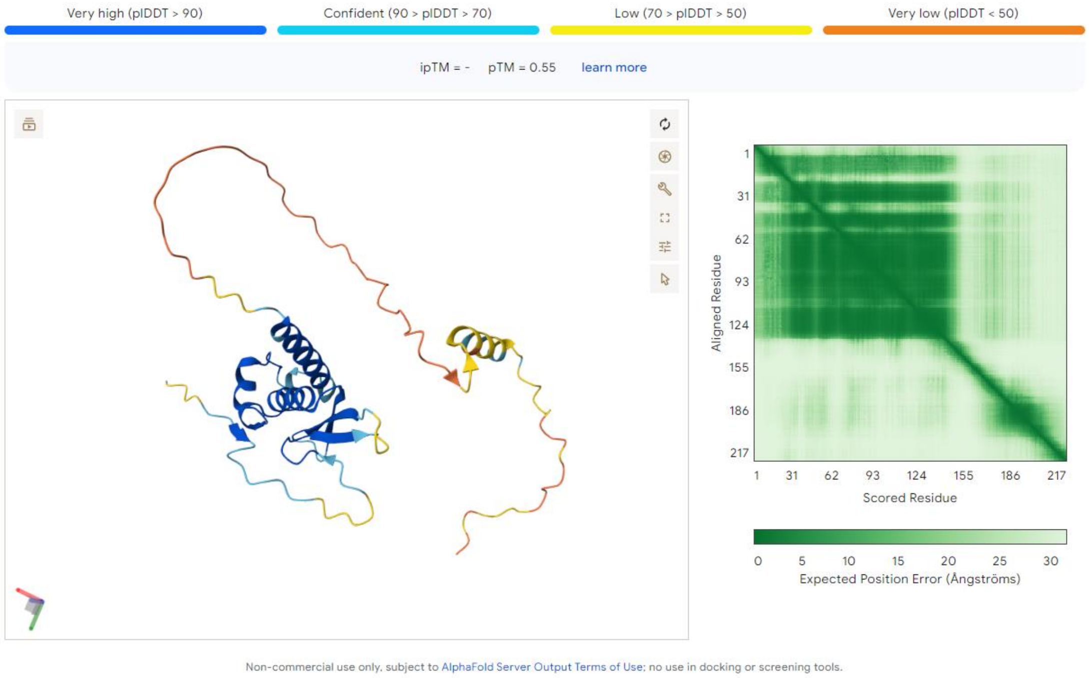

Information

Type	Copies	Sequence
Protein	1	MEVEEAAYRT (10) VWSEPPKRPA (20) GRTKFRETRH (30) PVYRGVRRRG (40) GRPGAAGRWV (50) CEVRVPGARG (60) SRLWLGTFAT (70) AEAAARAHDA (80) AALALRGRAA (90) CLNFADSAWR (100) MPPVPASAAL (110) AGARGVRDAV (120) AVAVEAFQRQ (130) SAAPSSPAET (140) FANDGDEEED (150) NKDVLPVAAA (160) EVFDAGAFEL (170) DDGFRFGGMD (180) AGSYYASLAQ (190) GLLVEPPAAG (200) AWWEDGELAG (210) SDMPLWSY (218)

Seed: 570892230

Figure S2. Quality and input information of the structure models of OsDREB1B generated using AlphaFold 3.

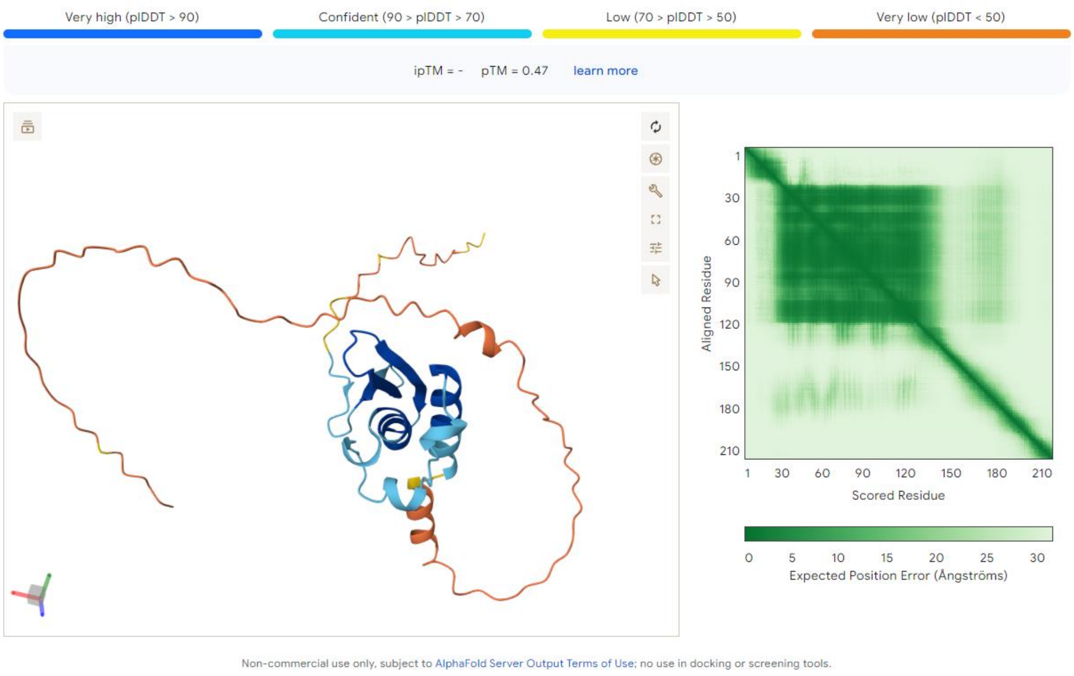

Information

Type	Copies	Sequence
Protein	1	MEYYEQEEYA (10) TVTSAPPKRP (20) AGRTKFRETR (30) HPVYRGVRRR (40) GPAGRWVCEV (50) REPNKKSRIW (60) LGTFATAEAA (70) ARAHDVAALA (80) LRGRGACLNF (90) ADSARLLRVD (100) PATLATPDDI (110) RRAAIELAES (120) CPHDAAAAAA (130) SSSAAAVEAS (140) AAAAPAMMMQ (150) YQDDMAATPS (160) SYDYAYYGNM (170) DFDQPSYYYD (180) GMGGGGEYQS (190) WQMDGDDDGG (200) AGGYGGGDVT (210) LWSY (214)

Seed: 232747348

Figure S3. Quality and input information of the structure models of OsDREB1C generated using AlphaFold 3.

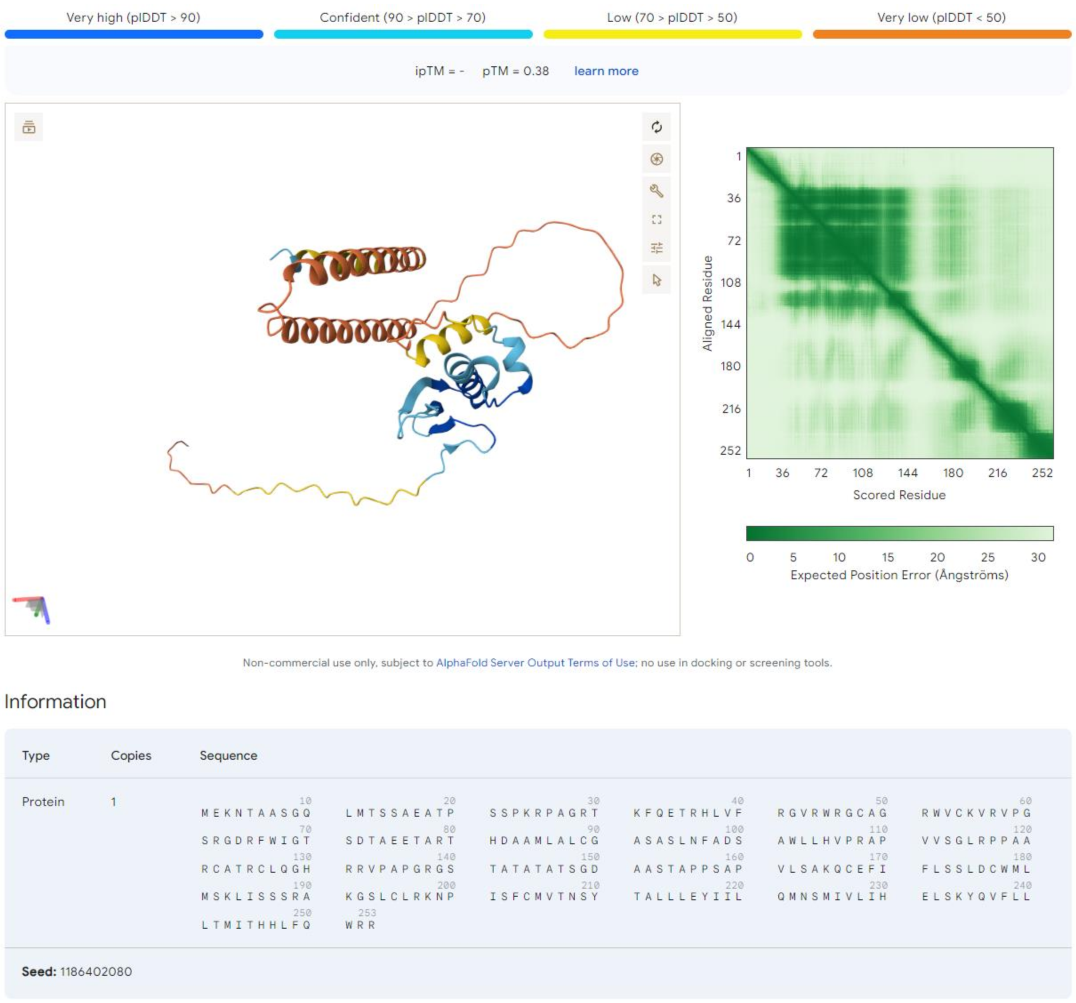

Figure S4. Quality and input information of the structure models of OsDREB1D generated using AlphaFold 3.

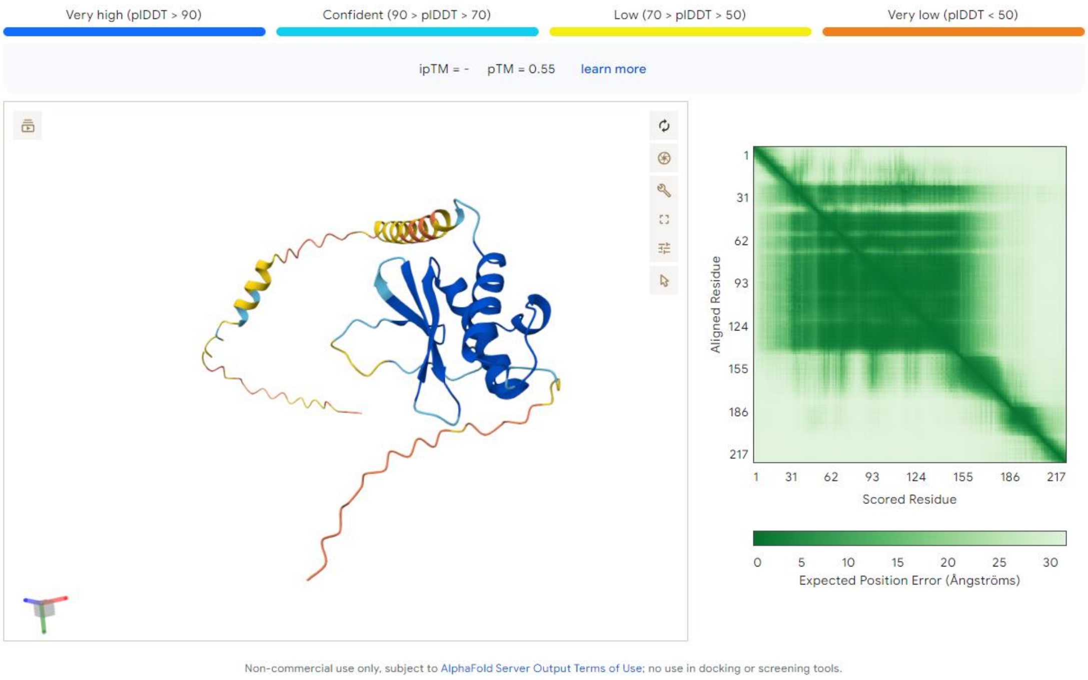

Information

Type	Copies	Sequence
Protein	1	MEWAYYGSGY SSSGTPSPVG GDGDEDSYMT VSSAPPKRRA GRTKFKETRH PVYKGVRSRN PGRWVCEVRE PHGKQRIWLG TFETAEMAAR AHDVAAMALR GRAACLNFAD SPRRLRVPPL GAGHEEIRRA AVEAAELFRP APGQHNAAAE AAAAVAAQAT AASAELFADF PCYPMDGLEF EMQGYLDMAQ GMLIEPPPLA GQSTWAEEDY DCEVNLWSY

Seed: 41750741

Figure S5. Quality and input information of the structure models of OsDREB1E generated using AlphaFold 3.

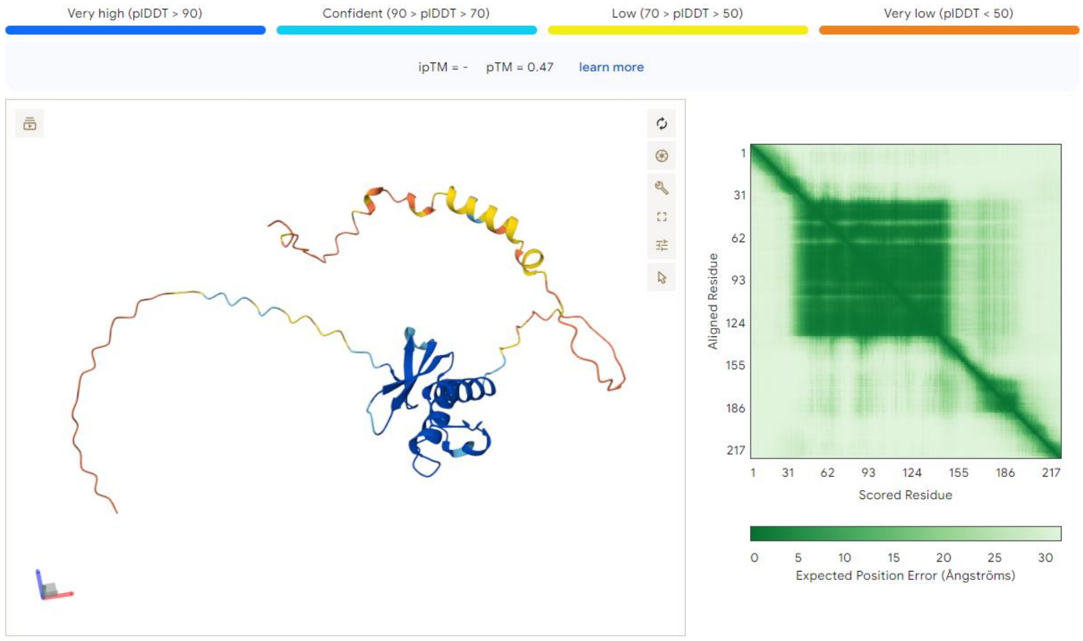

Non-commercial use only, subject to AlphaFold Server Output Terms of Use; no use in docking or screening tools.

Information

Type	Copies	Sequence
Protein	1	MDTEDTSSAS[10] SSSVSPPSSP[20] GGGHHHRLPP[30] KRRAGRKKFR[40] ETRHPVYRGV[50] RARAGGSRWV[60] CEVREPQAQA[70] RIWLGTYPTP[80] EMAARAHDVA[90] AIALRGERGA[100] ELNFPDSPST[110] LPRARTASPE[120] DIRLAAAQAA[130] ELYRRPPPPL[140] ALPEDPQEGT[150] SGGGATATSG[160] RPAAVFVDED[170] AIFDMPGLID[180] DMARGMMLTP[190] PAIGRSLDDW[200] AAIDDDDDHY[210] HMDYKLWMD[219]

Seed: 1228883957

Figure S6. Quality and input information of the structure models of OsDREB1F generated using AlphaFold 3.

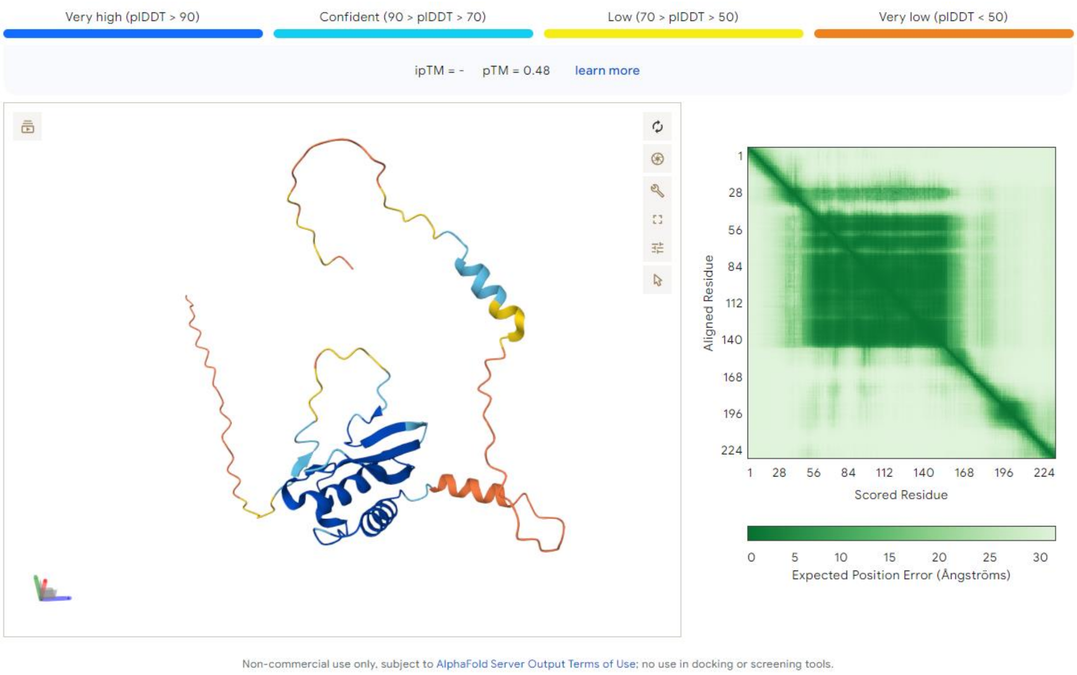

Information

Type	Copies	Sequence
Protein	1	MDVSAALSSD YSSGTPSPVA ADADDGSSAY MTVSSAPPKR RAGRTKFKET RHPVFKGVRR RNPGRWVCEV REPHGKQRIW LGTFETAEMA ARAHDVAALA LRGRAACLNF ADSPRRLRVP PIGASHDDIR RAAAEAAEAF RPPPDESNAA TEVAAAASGA TNSNAEQFAS HPYYEVMDDG LDLGMQGYLD MAQGMLIDPP PMAGDPAVGS GEDDNDGEVQ LWSY

Seed: 700090856

Figure S7. Quality and input information of the structure models of OsDREB1G generated using AlphaFold 3.

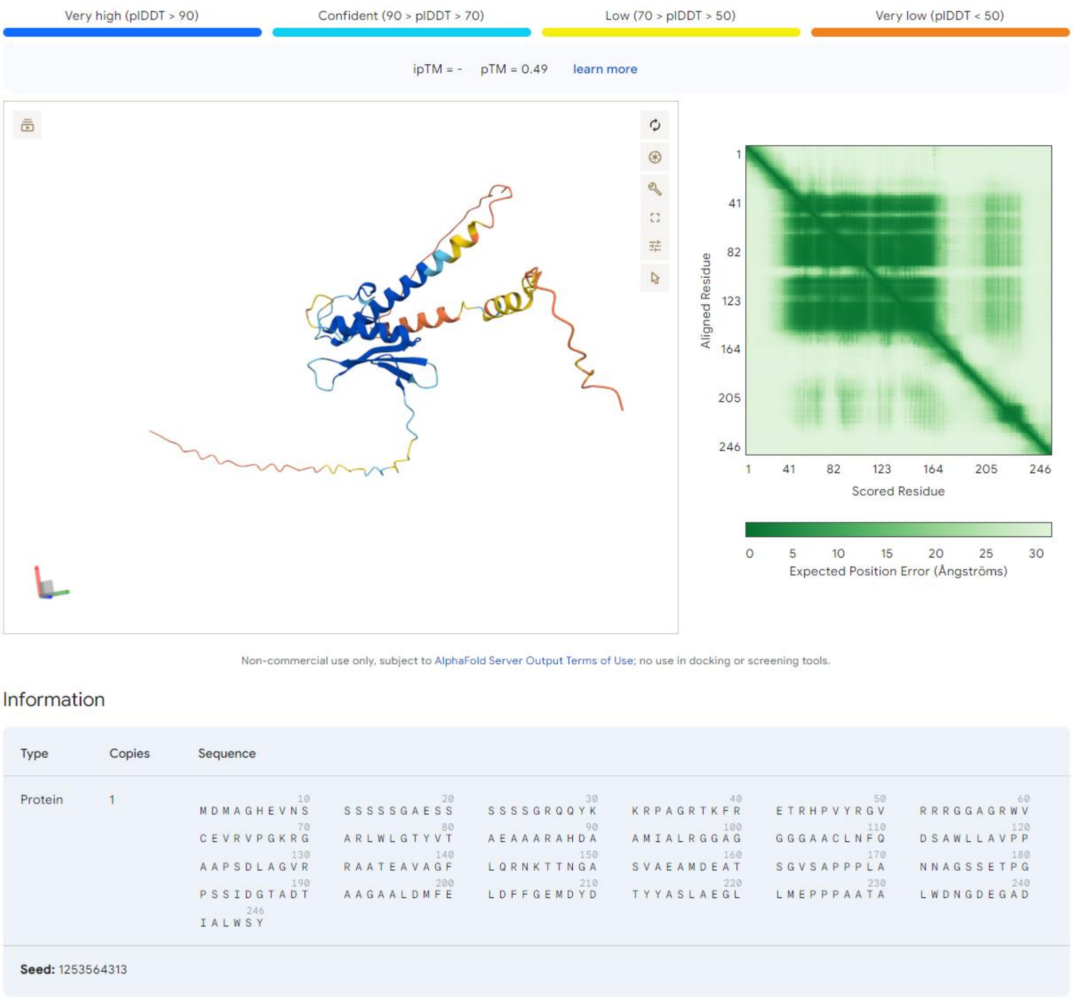

Figure S8. Quality and input information of the structure models of OsDREB1H generated using AlphaFold 3.

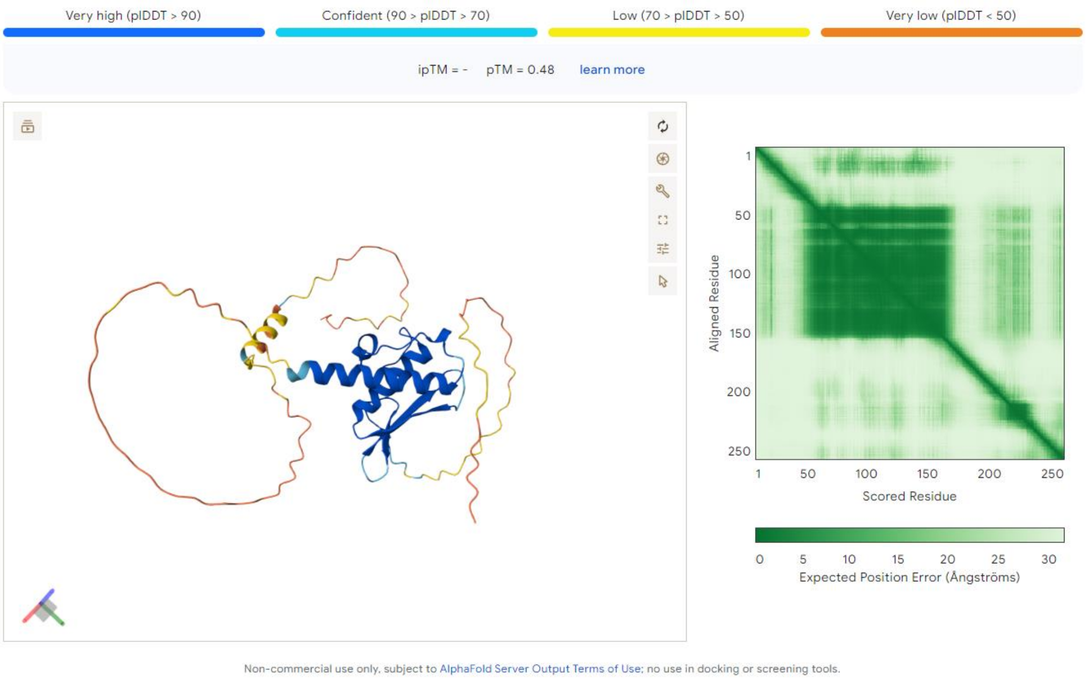

Information

Type	Copies	Sequence
Protein	1	MCTSKLEEIT GEWPPPALQA ASTTSSSEPC RRLSPPSSKR PAGRTKFHET RHPVFRGVRR RGRAGRWVCE VRVPGRRGCR LWLGTFDAAD AAARAHDAAM LALRGRAAAC LNFADSAWLL AVPPPATLRC AADVQRAVAR ALEDFEQRES SSSVFPLAID VVAEDAMSAT SEPSAASDDD AVTSSSSTTD ADEEASPFEL DVVSDMGWSL YYASLAEGLL MEPPASGASS DDDDDAIVDS SDIADVSLWS Y

Seed: 1070414207

Figure S9. Quality and input information of the structure models of OsDREB1I generated using AlphaFold 3.

Very high (pIDDT > 90) Confident (90 > pIDDT > 70) Low (70 > pIDDT > 50) Very low (pIDDT < 50)

ipTM = - pTM = 0.49 learn more

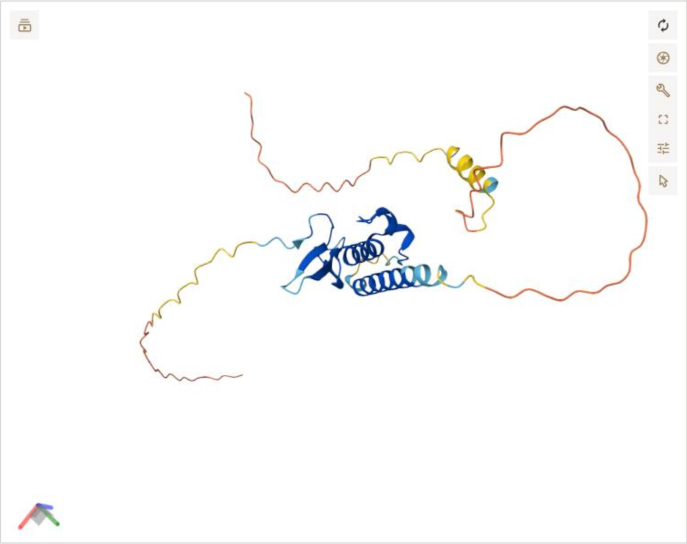

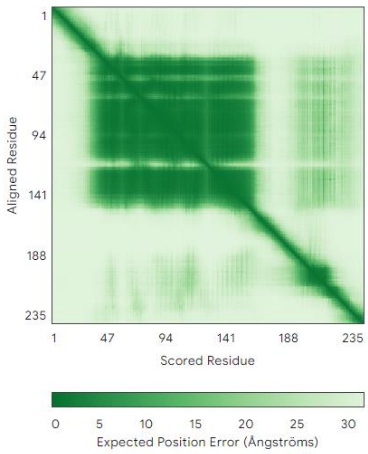

Non-commercial use only, subject to AlphaFold Server Output Terms of Use; no use in docking or screening tools.

Information

Type	Copies	Sequence
Protein	1	(see below)

10	20	30	40	50	60
MEKNTTAMGQ	LMSSSATTAA	TATGPASPKR	PAGRTKFQET	RHPVFRGVRR	RGRAGRWVCE
70	80	90	100	110	120
VRVPGSRGDR	LWVGTFDTAE	EAARAHDAAM	LALCGASASL	NFADSAWLLH	VPRAPVASGH
130	140	150	160	170	180
DQLPDVQRAA	SEAVAEFQRR	GSTAATATAT	SGDAASTAPP	SSSPVLSPND	DNASSASTPA
190	200	210	220	230	236
VAAALDHGDM	FGGMRTDLYF	ASLAQGLLIE	PPPPPTTAEG	FCDDEGCGGA	EMELWS

Seed: 1855804680

Figure S10. Quality and input information of the structure models of OsDREB1J generated using AlphaFold 3.

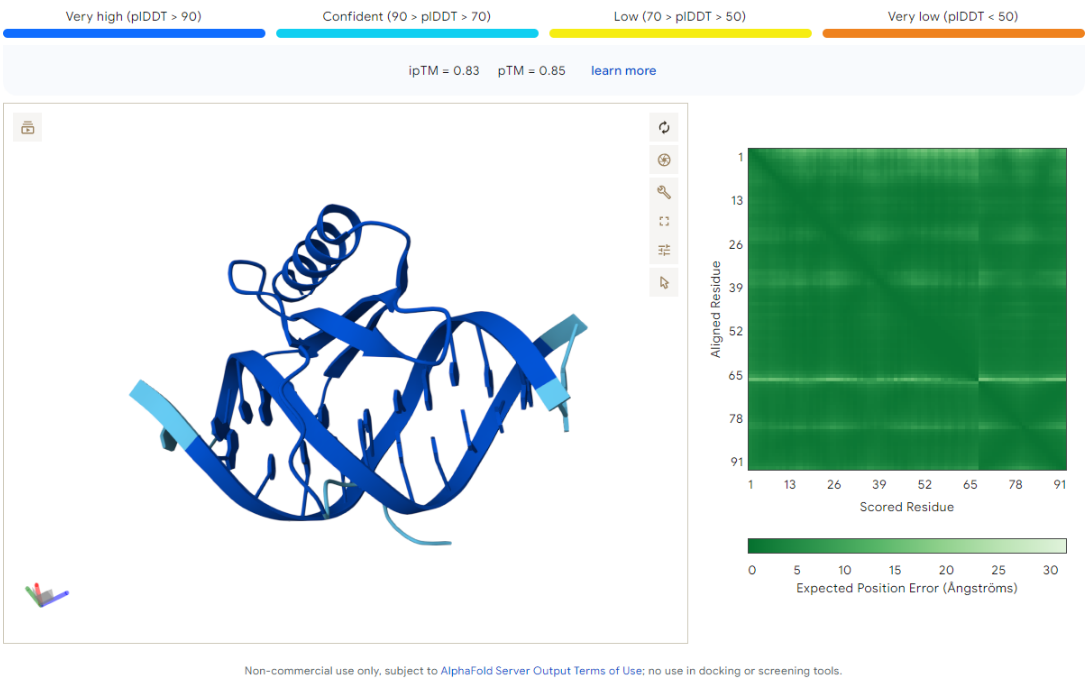

Information

Type	Copies	Sequence
Protein	1	KRPAGRTKFR ETRHPVFRGV RRRGNAGRWV CEVRVPGRRG CRLWLGTFDT AEGAARAHDA AMLAINAG
DNA	1	GTATGCCGAC ACT
DNA	1	AGTGTCGGCA TAC

Seed: 1826745704

Figure S11. Quality and input information of the complex structure models of OsDREB1A-DBD binding with DNA containing CRT element generated using AlphaFold 3.

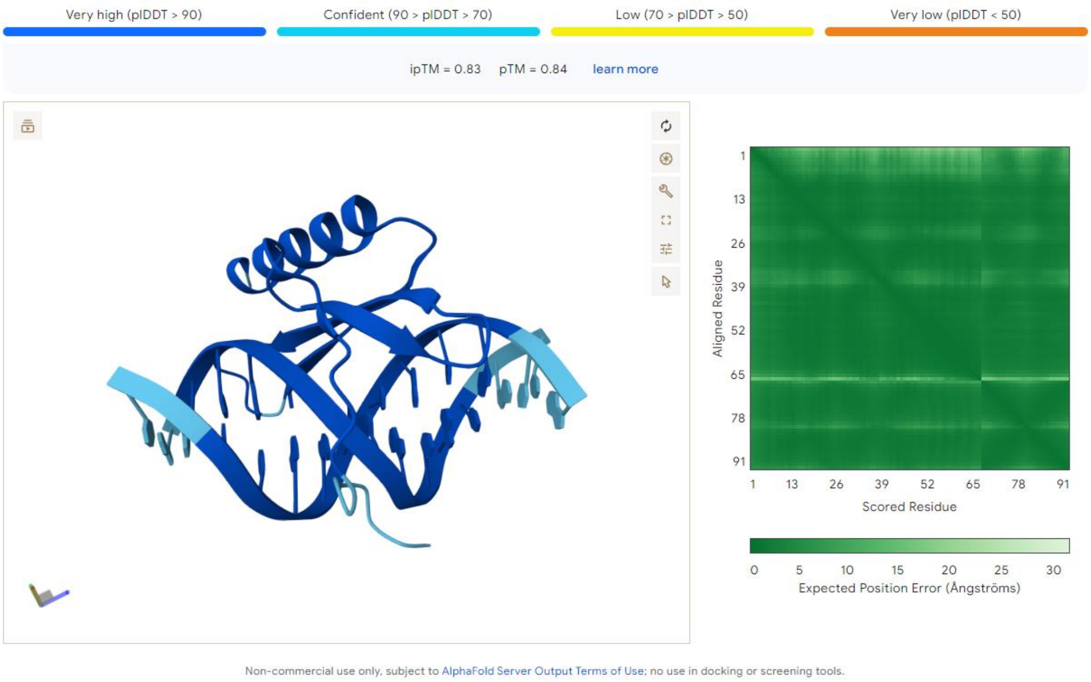

Information

Type	Copies	Sequence
Protein	1	KRPAGRTKFR ETRHPVFRGV RRRGNAGRWV CEVRVPGRRG CRLWLGTFDT AEGAARAHDA AMLAINAG
DNA	1	GTATACCGAC ACT
DNA	1	AGTGTCGGTA TAC

Seed: 1826745704

Figure S12. Quality and input information of the complex structure models of OsDREB1A-DBD binding with DNA containing DRE element generated using AlphaFold 3.

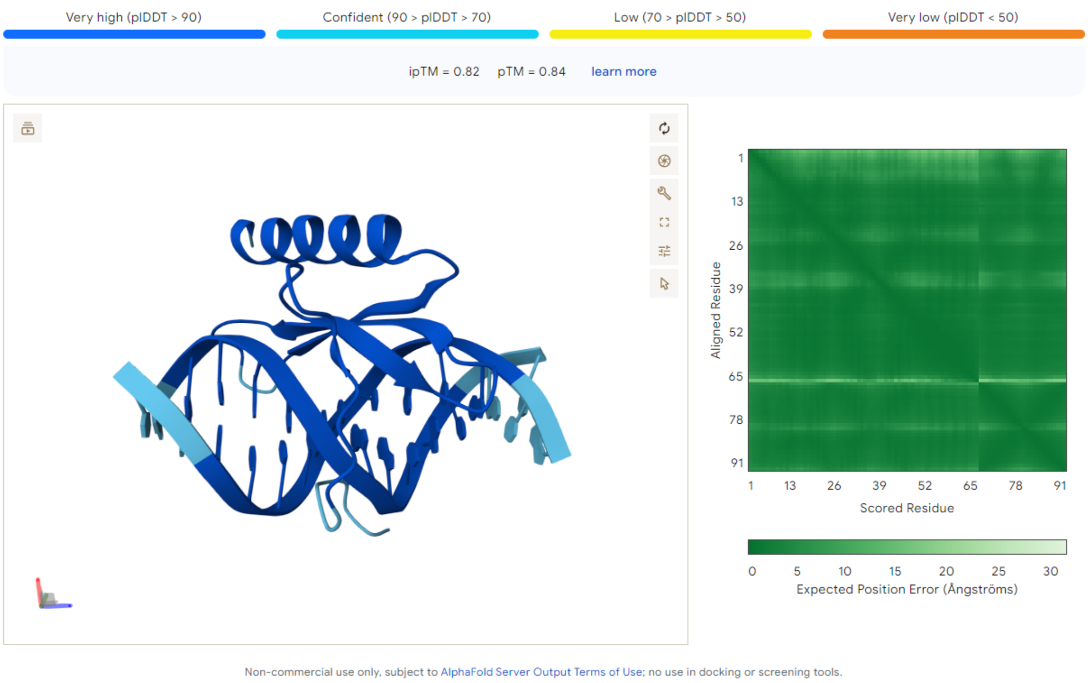

Information

Type	Copies	Sequence
Protein	1	KRPAGRTKFR ETRHPVFRGV RRRGNAGRWV CEVRVPGRRG CRLWLGTFDT AEGAARAHDA AMLAINAG
DNA	1	GTACGCCGCC ACT
DNA	1	AGTGGCGGCG TAC

Seed: 1826745704

Figure S13. Quality and input information of the complex structure models of OsDREB1A-DBD binding with DNA containing GCC-box element generated using AlphaFold 3.

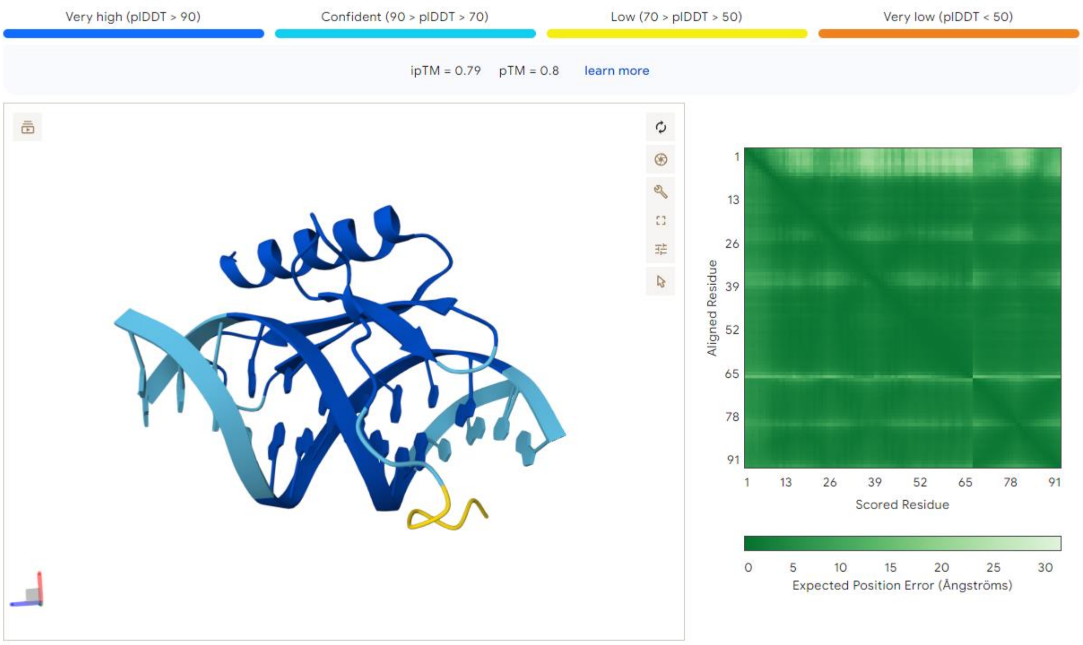

Non-commercial use only, subject to AlphaFold Server Output Terms of Use; no use in docking or screening tools.

Information

Type	Copies	Sequence
Protein	1	KRPAGATKFR ETRHPVFRGV RRRGNAGRWV CEVRVPGRRG CRLWLGTFDT AEGAARAHDA AMLAINA (10, 20, 30, 40, 50, 60, 67)
DNA	1	GTACGCCGAC ACT (10, 13)
DNA	1	AGTGTCGGCG TAC (10, 13)

Seed: 1826745704

Figure S14. Quality and input information of the complex structure models of OsDREB1A-DBD R40A binding with DNA containing CRT element generated using AlphaFold 3.

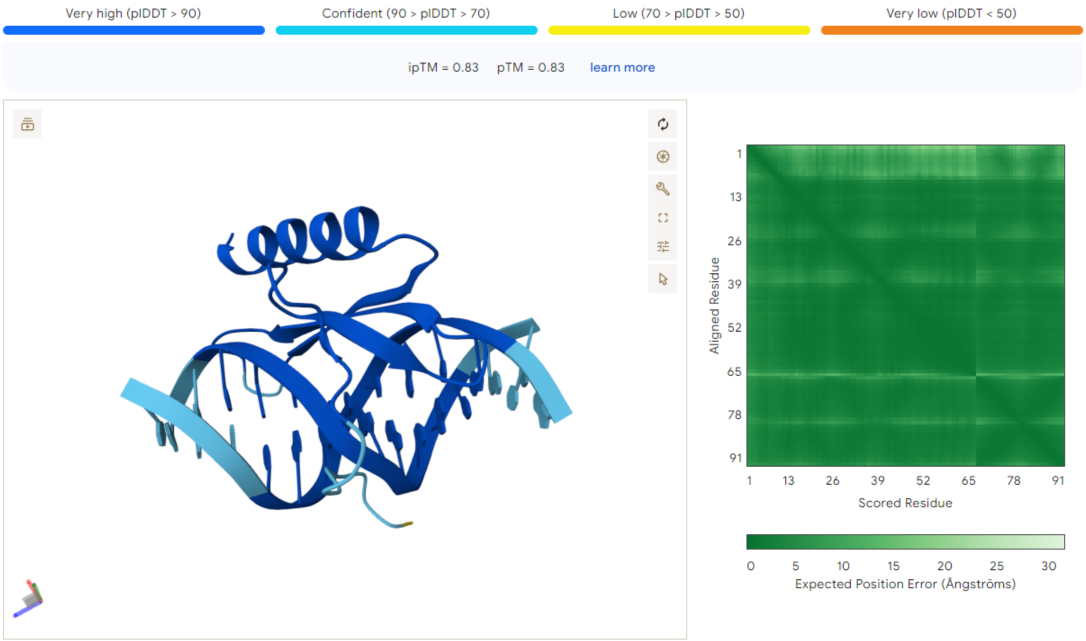

Non-commercial use only, subject to AlphaFold Server Output Terms of Use; no use in docking or screening tools.

Information

Type	Copies	Sequence
Protein	1	KRPAGRTKGR ETRHPVFRGV RRRGNAGRWV CEVRVPGRRG CRLWLGTFDT AEGAARAHDA AMLAINA
DNA	1	GTACGCCGAC ACT
DNA	1	AGTGTCGGCG TAC

Seed: 1826745704

Figure S15. Quality and input information of the complex structure models of OsDREB1A-DBD F43G binding with DNA containing CRT element generated using AlphaFold 3.

Very high (pIDDT > 90) | Confident (90 > pIDDT > 70) | Low (70 > pIDDT > 50) | Very low (pIDDT < 50)

ipTM = 0.83 pTM = 0.85 learn more

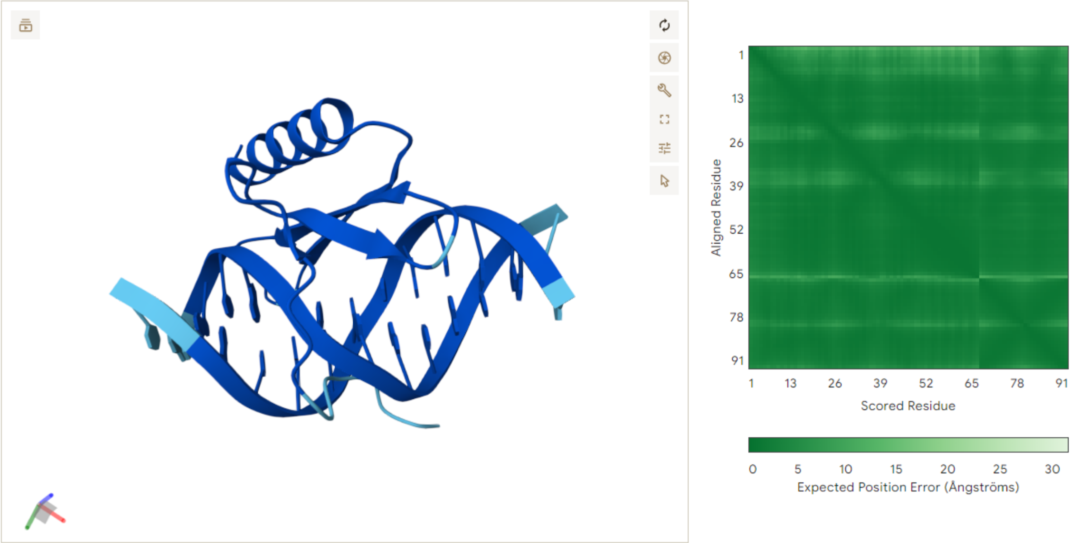

Non-commercial use only, subject to AlphaFold Server Output Terms of Use; no use in docking or screening tools.

Information

Type	Copies	Sequence
Protein	1	KRPAGRTKFR[10] ETRHPVFRGV[20] RRRGNAGRWA[30] CEVRVPGRRG[40] CRLWLGTFDT[50] AEGAARAHDA[60] AMLAINA[67]
DNA	1	GTACGCCGAC[10] ACT[13]
DNA	1	AGTGTCGGCG[10] TAC[13]

Seed: 1826745704

Figure S16. Quality and input information of the complex structure models of OsDREB1A-DBD V64A binding with DNA containing CRT element generated using AlphaFold 3.

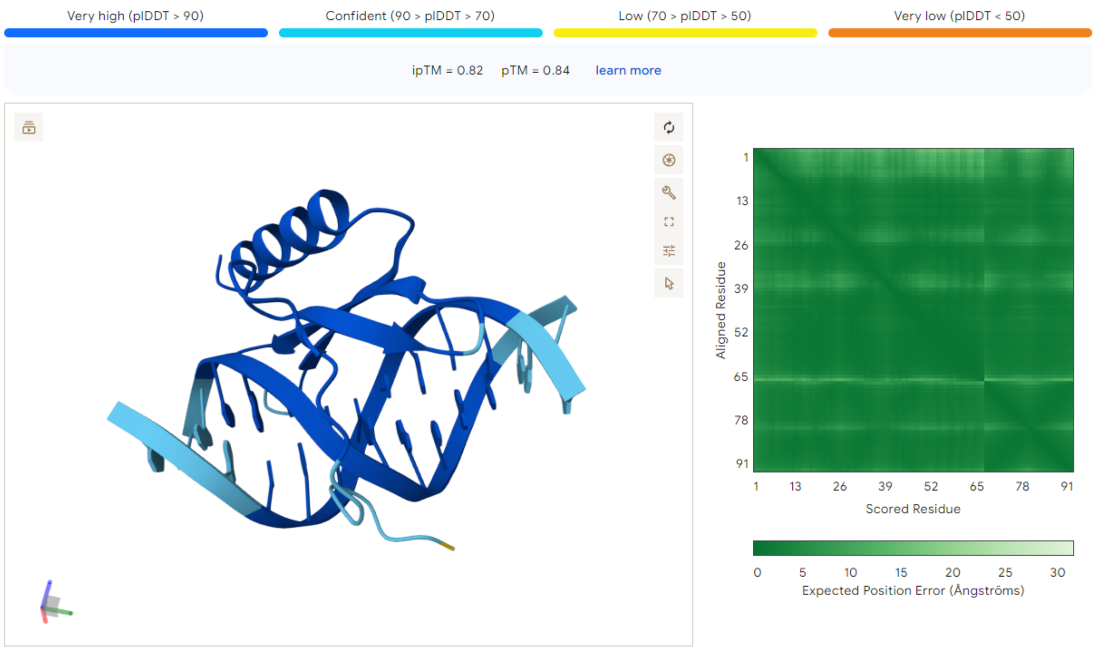

Non-commercial use only, subject to AlphaFold Server Output Terms of Use; no use in docking or screening tools.

Information

Type	Copies	Sequence
Protein	1	KRPAGRTKFR ETRHPVFRGV RRRGNAGRWV CAVRVPGRRG CRLWLGTFDT AEGAARAHDA AMLAINA
DNA	1	GTACGCCGAC ACT
DNA	1	AGTGTCGGCG TAC

Seed: 1208592968

Figure S17. Quality and input information of the complex structure models of OsDREB1A-DBD E66A binding with DNA containing CRT element generated using AlphaFold 3.
